# Supplementary material for: Scanning and active sampling behaviours emerge from conserved insect neural circuits
Source: eLife. 2026 Oct 6;15:RP110165. doi: 10.7554/eLife.110165 (PMC13641763; doi:10.7554/eLife.110165)
Supplement: Peer review history

# Editor report: eLife Assessment

**DOI:** 10.7554/eLife.110165.4.sa0

**Authors:** Albert Cardona

**Affiliations:** University of Cambridge United Kingdom

**Keywords:** Convincing, Important

A computational model potentially provides **important** new insights into the circuit mechanisms underlying navigational control in insects. The authors compare high speed video recordings of ants with detailed predictions from a new computational model. The similarities between model and behavioral data are striking and **convincingly** suggest how complex behavioral motifs can emerge from a simple neural circuit.

# Referee report: Reviewer #2 (Public review)

**DOI:** 10.7554/eLife.110165.4.sa1

The paper by Freas and Wystrach is an interesting computational study, exploring the detailed mechanisms of how simple neural circuits could explain complex behavioral patterns observed in navigating ants. The authors compare detailed, high speed video recordings of Australian desert ants (Melophorus bagoti) with predictions made by their new computational model and find convincing similarities between the model and the behavioral data, at a level of detail not previously studied. Particularly interesting are emerging properties of the model, yielding behavioral motifs it was not designed to reproduce, but which occur in natural ant behavior.

A strength of the study is that the model is based on previous models, without making major novel assumptions. It combines existing models of the insect central complex with a model of the lateral accessory lobe and adds a stochastic inhibition of forward velocity to the interaction of central complex and lateral accessory lobes. In essence, the central complex provides corrective steering signals when the goal direction and the current heading of the insect are not aligned, while the lateral accessory lobes provide an intrinsic oscillator underlying the behavioral oscillations shown by walking ants at all times. These background oscillations are modulated by the steering signals from the central complex. Depending on which phase of the intrinsic oscillations coincides with the corrective signals, and how fast the ant is moving forward during this time, a complex set of behaviors emerges.

Most prominently, scanning behaviors, which are regularly carried out by the ants, are recapitulated in great detail by the model. Additionally, other behaviors, such as full loops, emerge naturally from the model. While computational models are not to be seen as definite evidence for any biological reality, they can provide strong support for particular neural implementations. The current study is an excellent example in that it provides evidence for a serial arrangement of central complex circuits upstream of the lateral accessory lobe circuits, modulated by speed regulating input. While the latter is hypothetical, it yields a clear hypothesis that can be validated by connectomics studies and functional work in the future.

The computational model is explained in detail and information about all model parameters is provided in an accessible way. The approach is thus transparent and reproducible, leaving it to the readers to assess the assumptions made in the model and how the studied complex behaviors emerge. This also provides the possibility to combine this new model with existing models to expand the scope and to more comprehensively capture the behavioral repertoire of ants, and insects in general.

Importantly, the study shows that even complex behavioral motifs do not require dedicated neural modules, but can rather emerge from the interplay of already known circuits - highlighting the efficiency of insect brains and possibly providing the path towards embodied hardware solutions of such circuits in autonomous agents.

# Author comment: Author response

**DOI:** 10.7554/eLife.110165.4.sa2

**Authors:** Cody A Freas, Antoine Wystrach

**Affiliations:** Centre National de la Recherche Scientifique Toulouse France; CNRS - University Paul Sabatier of Toulouse Toulouse France

The following is the authors’ response to the previous reviews

**Public Reviews:**

**Reviewer #1 (Public review):**

Freas and Wystrach present a computational and experimental study of ant navigation. The main innovation of the computational model is the insertion of an oscillatory element between the steering signal and the motor control that results in a trajectory whose heading oscillates around a goal direction. Additionally, the model imposes periodic cessations of forward movement and inversely couples rotational speed to forward velocity. As a result the model periodically makes larger reorientations reminiscent of those seen in behaving ants.

The behavioral data consists of two experimental sets: experienced Melophorus bagoti foragers, recorded in 2010 and inexperienced M. bagoti foragers, recorded in 2023-2024 at the same site. The behavioral data is qualitatively compared to the model in Figures 3 through 6. In figures 3-5, all ant sets are grouped together while in Figure 6 they are separated. In Figure 6, the authors should do a careful job of making sure the reader is aware that comparisons are being made between behavioral data sets captured more than a decade apart and of justifying the validity of a quantitative comparison between these sets.

We now make explicit in the methods and figure that the two datasets were recorded at different times: experienced foragers from 2010 (Deeti et al., 2023) and inexperienced foragers from 2023. Their comparison is used to test a qualitative difference predicted by the model.

The manuscript also describes Myrmecia ants and makes comparisons between modeled Myrmecia ants and supplemental videos of these ants (Videos 3,4). These videos are not described in the methods. While the captions describe these as ants "homing in an unfamiliar environment," the videos show tethered ants walking on a ball. Without more information and absent any analysis, it is difficult for me to understand how these videos support granular points in the text about coupling between rotation and forward velocities.

We have added a description of Videos 3 and 4 to their respective captions and now state explicitly that these clips were recorded from ants on a tethered trackball apparatus and that they provided as qualitative examples of behaviour discussed in the text.

Strengths:

The manuscript's main thesis, that an oscillatory element interspersed between the control signal and the motor unit can reproduce aspects of ant navigation, appears supportable.

Weaknesses:

Qualitative agreement between aspects of a model and aspects of a behavioral measurement do not prove the correctness of a model. In the section (802), "An ancestral design? Striking parallels with crawling *Drosophila* larvae," the authors argue that behavioral data in larvae support their model, despite the larva's lack of a (known) central complex. *C. elegans* navigation can also be segmented into longer runs and shorter exploratory behaviors (Chen 2025), comparable to the runs and scans described here. *C. elegans* definitively does not have a central complex. In general, multiple internal mechanisms are capable of producing the same macroscopic behavioral outcome. This fact limits the ability of behavioral data to confirm the details of a particular model; it does not imply that observation of similar behaviors in multiple species shows that a particular model is correct or generalizable.

Here the ability of the behavioral data to confirm or constrain the model is further limited by the qualitative nature of the comparisons. Some of the comparisons are trivial (e.g. Figure 5E-F: any first order process will produce a Poisson distribution, and in the model a Poisson process was explicitly coded in with parameters chosen (1070) to match the behavioral data). Finally, the number of adjustable parameters (13) is comparable to the number of comparisons made; it is unclear that the model could not be adjusted to fit any set of behavioral measurements.

Our model is a minimal neuro-mechanical model. It is not a mathematical model where each parameter can be optimised to a final output.

From our 13 parameters, 10 were either taken directly from independent prior studies. 5 concern the oscillator, and have been arbitrarily chosen and simply need to produce regular oscillation (as explained in supplemental material). 4 are the necessary motor gain and noise, which scale neural activations values into movement units, note that this conversion is backed up by previous evidence in *Drosophila* and present in previous ant models. 1 parameter specifies the width of the bump of activity in the CX, and is roughly matched to neural imaging data in flies. None of these parameters have been introduced or adjusted to back up our claim.

Only 3 parameters have been added to produce scannings. Two of them were tuned to match local scanning-specific data (the probabilistic trigger to stop (p_stop) and the threshold for triggering a saccade (θ_CPG) enabling us to tune fixation duration). This level of parametrisation enables the model to reproduce realistic scans, but does not influence the qualitative predictions of this article. For instance, we agree that the probabilistic trigger producing the Poisson distribution of scan duration (Figure 5’s E) is used to parametrise the model to scanning data, which does not constitute an emerging prediction of the model. We do not include it as evidence (see ~490). Finally, the CX_output_gain, is a new parameter we invoke to implement our hypothesis that CX steering modulates the oscillator. From these three added parameters emerge the large array of behavioural signatures and predictions. These are emerging consequences of the model's architecture rather than curve-fits.

While the introduction is improved, there is still room to eliminate confusion as to what aspects of the model reflect hypothesized rather than measured neural circuits. For instance, if there is data showing LAL oscillations in insects, the authors should cite it and call it out clearly.

Alternatively they should say that the oscillator is hypothesized based on measured bistability. They should also clarify whether they are discussing neural oscillations or motor oscillations and whether these oscillations are measured, modeled, or hypothesized.

As one example: Lines 283-284 "This oscillator [referring to the model's intrinsic oscillator described in the previous paragraph], which is widespread in insects (Cheng, 2024; Kanzaki, 2005; Kanzaki and Mishima, 1996), resides in the lateral accessory lobes (LAL)" reads as though it is known that a neural oscillator occupies the LAL. Cheng 2024 is a brief review of behavioral oscillation. Kanzaki et al. 2005 describes numerical modeling and simulation with a physical robot. Kanzaki and Mishima, 1996 demonstrates bistability (flip-flopping) in moth descending neurons. None of these show neural oscillations and none of them describe the LAL. The authors should review the paper and be scrupulously careful that the claims made in the text are supported in the cited references. These difficulties were pointed out in a previous round of review; hopefully they can be fully corrected this time.

Kevin S. Chen, Jonathan W. Pillow*, Andrew M. Leifer*, "State-switching navigation strategies in *C. elegans* are beneficial for chemotaxis," arXiv:2508.00191 31 July 2025.

We have softened the text to be more explicit about what is modelled versus what is neurally shown (labelled each as behavioural, electrophysiological, or modelled)

**Reviewer #2 (Public review):**

The paper by Freas and Wystrach is an interesting computational study, exploring the detailed mechanisms of how simple neural circuits could explain complex behavioral patterns observed in navigating ants. The authors compare detailed, high speed video recordings of Australian desert ants (Melophorus bagoti) with predictions made by their new computational model and find convincing similarities between the model and the behavioral data, at a level of detail not previously studied. Particularly interesting are emerging properties of the model, yielding behavioral motifs it was not designed to reproduce, but which occur in natural ant behavior.

A strength of the study is that the model is based on previous models, without making major novel assumptions. It combines existing models of the insect central complex with a model of the lateral accessory lobe and adds a stochastic inhibition of forward velocity to the interaction of central complex and lateral accessory lobes. In essence, the central complex provides corrective steering signals when the goal direction and the current heading of the insect are not aligned, while the lateral accessory lobes provide an intrinsic oscillator underlying the behavioral oscillations shown by walking ants at all times. These background oscillations are modulated by the steering signals from the central complex. Depending on which phase of the intrinsic oscillations coincides with the corrective signals, and how fast the ant is moving forward during this time, a complex set of behaviors emerges.

Most prominently, scanning behaviors, which are regularly carried out by the ants, are recapitulated in great detail by the model. Additionally, other behaviors, such as full loops, emerge naturally from the model. While computational models are not to be seen as definite evidence for any biological reality, they can provide strong support for particular neural implementations. The current study is an excellent example in that it provides evidence for a serial arrangement of central complex circuits upstream of the lateral accessory lobe circuits, modulated by speed regulating input. While the latter is hypothetical, it yields a clear hypothesis that can be validated by connectomics studies and functional work in the future.

The computational model is explained in detail and information about all model parameters is provided in an accessible way. The approach is thus transparent and reproducible, leaving it to the readers to assess the assumptions made in the model and how the studied complex behaviors emerge. This also provides the possibility to combine this new model with existing models to expand the scope and to more comprehensively capture the behavioral repertoire of ants, and insects in general.

Importantly, the study shows that even complex behavioral motifs do not require dedicated neural modules, but can rather emerge from the interplay of already known circuits - highlighting the efficiency of insect brains and possibly providing the path towards embodied hardware solutions of such circuits in autonomous agents.

We thank Reviewer 2 for this assessment.

**Recommendations for the authors:**

**Reviewer #1 (Recommendations for the authors):**

The paper would benefit if the authors would take a more traditional/formal approach to the presentation and interpretation of results. They should avoid drawing conclusions or presenting interpretations in the figure captions (e.g. caption to figure 6) and avoid unnecessary modifiers (e.g. just use "supports" instead of "strongly supports"). This might help correct the tendency of the manuscript to overstate or over-interpret the correspondence between the model and the data.

Figure captions are now revised to remove interpretive discussion. We also removed unnecessary modifiers throughout the manuscript.

We also added clarifying text to the “An ancestral design?” section to make clear that we are not implying homologous neural implementations across taxa.

**Reviewer #2 (Recommendations for the authors):**

The authors have addressed my comments fully and I only have a few minor, mostly editorial points:

line 142: it appears that the references should refer to goal encoding, but both references are head direction papers (one review, one research paper). The only paper showing goal encoding in the CX is Mussels-Pires et al 2024. This should be fixed to ensure the citations are not misleading.

Changed citations.

line 165: maybe remove 'intrinsic' to not suggest that this reflects what it known from Biology? It is clear that in the model it is an intrinsic oscillator, but it should not leave the impression that this is an established fact for the LAL

Removed when not discussing the model.

line 168: remove either 'a diversity' or 'key qualitative'

Changed to reproduce multiple key qualitative…. (~Line 170)

section: 'Neural substrate of insect navigation':

- '... compares to output steering commands.' grammar is misleading as 'output' might be read as an adjective rather than a verb, replace with 'generate'?

Changed.

- as above, the only paper showing goal directions is Mussels-Pires et al 2024. Any other paper either assumes this in models (such as Stone et al and the Honkanen review) or deal with head direction encoding. Pfeiffer and Homberg, 2014 is a general review. Please ensure that references are used more accurately.

We revised the text to clarify the specific evidence provided by each cited reference.

- The goal direction in the CX can be updated by various pathways....' After this, behavioral and modeling papers are cited, which is misleading. None of these papers deal with the CX or the neural representation of goals. Same with the rest of the sentence, referring to MB output and PI, which is only shown in modeling, not data.

We now explicitly distinguish between behavioural evidence and modelling evidence.

line 210: use CX, not central complex

Changed.

Figure 2: I suppose all data shown are modeling data? This should be more explicit in the figure caption (a bit unclear what 'using the neural circuit model).' in the caption heading means. Maybe rephrase to: 'Schematic of neural circuit model and its outputs across navigation relevant brain regions.' (or something like that, putting model first, not brain regions)

Changed.

Figure 7: Axis labels in the graphs are still much too small to be read on a printed version (ensure at least 5pt font size in the actual figure on the printed page)

Enlarged axis labels.

Line 654: mirror, not mirrors

Changed.

line 816: insert 'fly' before larva, as otherwise one might assume this refers to ant larva

Added (~line 822).

line 819: The CX does (as we currently know) not exist in fly larvae. At least not as a brain structure, or a set of homologous neurons. There might be equivalent circuits for action selection, but they have not yet been convincingly described. I suggest to rephrase to: 'Although no present as neuropils in fly larvae, the CX and LAL....'

Changed as suggested (~Line 830).
